# From strain to estrangement: the role of personality in predicting parent-child relational outcomes

**DOI:** 10.3389/fpsyg.2026.1859494

**Published:** 2026-06-22

**Authors:** Jag S. Gill, Matthias J. Barker, Carl P. Nienhuis

**Affiliations:** 1 Clinical Psychology, Adler University, Vancouver, BC, Canada; 2 Independent Researcher, Nashville, TN, United States; 3 Faculty of Health Sciences, University of the Fraser Valley, Chilliwack, BC, Canada

**Keywords:** estrangement, family stress, Five-Factor Theory, parent-child estrangement, parent-child relationships, personality

## Abstract

**Introduction:**

Parent-child estrangement is a prevalent yet understudied phenomenon, with existing research largely emphasizing circumstantial and cultural explanations while overlooking the potential role of personality. Guided by Five-Factor Theory, the present study examined whether Big Five personality traits and their lower-order aspects predict estrangement outcomes among parents and adult children within strained relational contexts.

**Methods:**

Using a cross-sectional survey design, participants (*N* = 520; 368 parents, 152 adult children) were recruited from an online parent-child estrangement support community and completed measures of demographic characteristics, estrangement status (operationalized via frequency of contact and communication), and personality (Big Five Aspects Scale). Binary logistic regression analyses were conducted separately for parents and adult children, controlling for relevant demographic variables.

**Results:**

Among parents, higher extraversion significantly predicted increased odds of being estranged by an adult child. Among adult children, higher openness significantly predicted reduced odds of initiating estrangement. No other personality traits were significant predictors in either group. With respect to demographic variables, higher educational attainment among adult children was associated with reduced odds of estrangement.

**Discussion:**

These findings suggest that personality traits help explain why similar relational contexts culminate in estrangement for some individuals but not others. Consistent with Five-Factor Theory, results support the proposition that basic tendencies are associated with characteristic adaptations, such as relational expectations, cognitive appraisals, and conflict styles, which are associated with estrangement-related behaviors. By identifying personality as a theoretically grounded and previously unexamined contributor, this study supports the development of more integrative models of estrangement that incorporate traits, culture, and circumstance.

## 1 Introduction

The parent-child bond is often considered one of the most cherished and enduring relationships, typically marked by emotional support, tangible resources, and frequent contact across the lifespan (Fingerman et al., 2009). For many, parent-child ties remain a source of stability and connection throughout the lifespan, with many considering their parent or child among their most important social ties (Hartnett et al., 2013; Van Gaalen and Dykstra, 2006). Yet, recent evidence shows that some parents and adult children deliberately choose to reduce or sever contact with one another, a phenomenon known as parent-child estrangement (Blake, 2017). In such cases, accumulating evidence indicates that estrangement can negatively affect the physical and emotional wellbeing of both parties, though particularly the individual being estranged (Reczek et al., 2024; Scharp, 2016). Estimates suggest that the lifetime prevalence rates of parent-child estrangement, depending on the measure used and the differences in data representativeness, range from 6% to 28% (Becker and Hank, 2022; Gilligan et al., 2015; Pillemer, 2020; Reczek et al., 2022).

Current conceptions of parent-child estrangement often focus on identifying circumstantial factors that cause estrangement, such as parental abuse or neglect, parental divorce, substance abuse, relational conflict, and undue influence of a therapist or another third party, such as an adult child's partner (Agllias, 2015, 2016; Blake, 2017; Blake et al., 2019; Conti, 2015; Scharp and McLaren, 2018; Schoppe-Sullivan et al., 2023). In response to such research, Reczek (2025) argued that while these circumstantial factors are undoubtedly relevant, many of them are neither necessary nor sufficient conditions for relational rupture. Numerous individuals who report exposure to such family dynamics do not experience estrangement, while others initiate estrangement in the absence of such family dynamics Reczek, (2025). As such, Reczek (2025) posited that this pattern suggests that circumstantial explanations alone cannot account for why estrangement occurs for some individuals but not others.

Alternatively, while building on Giddens (1992) theory of “pure relationship,” Reczek (2025) argued that estrangement decisions, while influenced by circumstances, are fundamentally shaped by culturally embedded “repertoires”– also referred to as schemas, frames, or scripts-that individuals draw upon to interpret and evaluate family relationships. More specifically, Reczek situated her argument historically by drawing on a period in the late 20th and early 21st centuries when family relationships were framed by a cultural repertoire known as “compulsory kinship.” This framework characterized parent-child ties as natural and forever enduring, colored with unconditional love and an emphasis on long-established social roles. However, she proposed that adult children have recently adopted a new cultural repertoire, primarily from exposure to new social networks, digital media, and therapy, that not only differs from their parents' generation but also directly conflicts with it. Reczek called this new cultural repertoire “democratized kinship,” emphasizing parent-child relationships rich in intentional connection, mutual respect, accountability, and emotional and physical safety. Reczek's (2025) qualitative interviews with 68 estranged adults in the United States demonstrated that adult children now view family relationships as conditional on these standards rather than obligatory or permanent. When family members are perceived as failing to meet these expectations, estrangement becomes a culturally legitimate course of action. Accordingly, Reczek (2025) understood estrangement not simply as a response to negative family experiences but as a decision guided by widely circulating and shifting cultural frameworks that shape beliefs about the conditions under which family ties should be sustained or discontinued.

While Reczek's (2025) framework usefully contextualized estrangement dynamics, it is vulnerable to the same critiques she applies to alternative circumstantial explanations of estrangement. That is, while conflicting beliefs and cultural norms within a family are undoubtedly relevant in understanding estrangement, they are not sufficient conditions for relational rupture. For instance, even when parents and adult children hold markedly different beliefs and expectations about what family relationships should require (e.g., compulsory vs. democratized kinship), relationships do not uniformly rupture. Many parents and adult children maintain contact despite value dissimilarity, political disagreements, or mismatched expectations of family roles (Gilligan et al., 2015; Hwang et al., 2024). This suggests that while the concept of “democratized kinship” provides a cultural lens for viewing and interpreting estrangement, it still does not explain why, despite similar parent-child dynamics and shared cultural frameworks, some ties culminate in estrangement while others do not.

A complementary framework incorporates not only the aforementioned family dynamics (both circumstantial causes and cultural repertoires), but also their interaction with stable individual personality traits that shape how relational experiences are interpreted and responded to over time. In this way, the Five-Factor Theory (FFT)-grounded in decades of factor-analytic research on patterns of covariation among personality characteristics-offers a critical explanatory component that has been entirely absent from existing estrangement research. By situating estrangement at the center of the interaction between enduring personality traits and circumstantial and cultural processes, FFT offers a culturally informed framework that explains why diverse and sometimes contradictory family circumstances culminate in estrangement for some individuals but not others. In short, FFT conceptualizes personality as a dynamic system composed of biologically rooted traits and environmental influences, including culture, that together guide behavior across contexts (McCrae and Costa, 2008).

At the core of the FFT are *basic tendencies*, including the widely researched Big Five traits of extraversion, agreeableness, conscientiousness, neuroticism, and openness (McCrae and Costa, 1999; Prinzie et al., 2009). Basic tendencies are understood as stable and heritable dispositions that reflect temperamental foundations for thinking, feeling, and behaving, influencing the broader personality system but not themselves directly shaped by experience or cultural context (McCrae and Costa, 2008; McCrae and Sutin, 2018). FFT holds that basic tendencies interact with *external influences*, such as cultural norms, life events, and interpersonal environments. Such an interaction creates *characteristic adaptations*, which are referred to as a range of acquired psychological structures, including values, goals, beliefs, coping strategies, habits, relational expectations, and internalized social roles (McCrae and Costa, 2008). Characteristic adaptations, in turn, then interact with specific external influences to produce manifestations of personality in the form of behaviors, thoughts, and feelings, ultimately resulting in what FFT refers to as one's *objective biography* (McCrae and Sutin, 2018; Mõttus, 2017). McCrae (2016) refers to the two aforementioned processes—(1) the creation of characteristic adaptations and (2) their manifestation into one's objective biography-as accommodation and assimilation processes, respectively. In other words, the accommodative process describes the development of characteristic adaptations (through the interaction between basic tendencies and external influences), and the assimilative process describes their expression in one's objective biography (i.e., through the interaction between characteristic adaptations and environmental influences) as behaviors and reactions (McCrae, 2016).

Applied to the present context, FFT offers a unifying framework that integrates circumstantial stressors, cultural repertoires, and stable individual personality traits into a coherent account of estrangement. Within FFT, Reczek's (2025) cultural repertoires emerge through an accommodative process, whereby individuals adapt their beliefs, values, and expectations about family (i.e., characteristic adaptations) through exposure to social networks, digital media, and therapy (i.e., external influences). Crucially, FFT specifies that this process is shaped by personality traits (i.e., basic tendencies), such that individuals differ in how they attend to, interpret, and incorporate these external influences. Consequently, exposure to similar external influences may produce divergent characteristic adaptations depending on underlying basic tendencies. Circumstantial stressors (i.e., external influences) then operate as proximal triggers that interact with these trait-shaped characteristic adaptations through an assimilative process, culminating in observable behavioral outcomes (i.e., objective biography), including estrangement. Thus, rather than viewing estrangement as driven primarily by circumstantial stressors or shifting cultural norms alone, FFT conceptualizes estrangement as the product of long-term interactions among enduring personality traits, culturally shaped repertoires, and situational pressures.

In this way, personality functions as a critical mechanism that explains why similar family environments and cultural contexts do not uniformly lead to estrangement. Building on this framework, existing personality research provides strong evidence for predicting how particular personality traits may shape an adult child's internalization of characteristic adaptations (e.g., democratized kinship) and, in turn, influence behavioral responses that are directly relevant to estrangement. With respect to accountability, agreeableness and conscientiousness are associated with greater forgiveness, empathy, emotional regulation, and constructive conflict engagement, possibly facilitating more generous interpretations of parental efforts at apologizing, whereas higher neuroticism is linked to rumination, emotional reactivity, and heightened threat sensitivity that may undermine confidence in parental accountability (Brose et al., 2005; Javaras et al., 2012; Koutsos et al., 2008; Melchers et al., 2016; Suls and Martin, 2005; Tehrani and Yamini, 2020). Perceptions of intentional connection are shaped by social motivation and reward sensitivity, with extraversion being associated with a stronger desire for frequent interaction and emotional expressiveness, such that more extraverted adult children may require greater relational engagement to experience relationships as satisfying (DeYoung, 2015; Lucas et al., 2008). The internalization of mutual respect is tied to tolerance of value differences, perspective-taking, and empathy, all of which are associated with higher agreeableness and openness, suggesting lower levels of these traits may influence whether similar parental behaviors are experienced as disrespectful or invalidating (Koutsos et al., 2008; Ng et al., 2021). Finally, evaluations of emotional and psychological safety are shaped by, among other factors, dispositional patterns of threat appraisal and stress regulation, with higher neuroticism being associated with heightened vigilance and emotional instability and lower neuroticism and higher conscientiousness supporting greater regulatory capacity and stress tolerance (Lahey, 2009; Luo et al., 2023).

Together, this body of work illustrates how an adult child's basic tendencies can shape the development of characteristic adaptations relevant to estrangement. Furthermore, given the inherently relational nature of estrangement, a focus on parental personality is also warranted. This is supported by two converging strands of research. First, a substantial body of evidence demonstrates that the Big Five traits influence parenting through their effects on parents' typical emotional states, cognitive interpretations, and behavioral tendencies (Malouff et al., 2010; Prinzie et al., 2009). Second, qualitative research consistently indicates that adult children frequently attribute their initiation of estrangement to enduring features of their parents' personalities. For example, Carr et al. (2015), in a study of 898 parents and adult children, found that adult children frequently cited parental personality as a primary reason for disengagement, often emphasizing its perceived resistance to change. Similarly, Scharp's (2019) analysis of narrative accounts from 52 estranged adult children identified personality-related characteristics as salient contributors to estrangement decisions. In addition, Blake's (2015) large-scale research project at the University of Cambridge Center for Family Research, in collaboration with Stand Alone-an organization that offers support services to estranged adults-reported that of the 807 participants in the study, approximately half cited a “clash of personality” as a factor contributing to their initiation of estrangement. More recently, Nikolajsen et al. (2025) have underscored the importance of accounting for personality in estrangement research due to its potential confounding effects.

Despite growing scholarly exploration of parent-child estrangement, no study to date has directly examined the role of personality traits in predicting estrangement outcomes. Existing research has focused primarily on circumstantial stressors and, more recently, cultural repertoires, yet the potential contribution of enduring dispositional differences remains entirely unexplored at the quantitative level. As such, this study's purpose was to address this gap by applying Five-Factor Theory to estrangement to investigate whether Big Five personality traits (and their facets) predict (a) whether an adult child has initiated estrangement from a parent, and (b) whether a parent has been estranged by an adult child.

Importantly, this study moves beyond traditional comparisons between intact, healthy families and estranged families-an approach that has informed much of the current literature-and instead focuses on individuals drawn from strained relational contexts. By examining variation within this sample, the present design offers a more specific exploration of whether stable personality traits are associated with divergent relational outcomes beyond shared circumstantial stressors. Moreover, because the sample is drawn from a U.S. context in which democratized kinship repertoires are culturally salient, this study also implicitly holds constant broader cultural narratives (Reczek, 2025). In doing so, this study represents an initial step toward developing a more specified explanatory model of estrangement that incorporates personality-based mechanisms.

## 2 Materials and methods

### 2.1 Participants and procedures

Participants were recruited from an online parent-child estrangement group (www.estrangement.com), founded by the second author in 2023. Participants in this group include parents and adult children experiencing strained family dynamics or estrangement. This online group offers workshops that provide tools and psychoeducation relevant to estrangement, as well as support groups for individuals seeking to connect with others experiencing strained relationships or estrangement. Blake et al. (2022) evaluated the utility of such estrangement support groups and found that they can reduce psychological distress associated with estrangement, as attendees often feel less alone and ashamed after meeting others in similar situations.

In October 2024, the second author sent an email to the approximately 2,000 global participants who had signed up for this group, inviting them to participate in an online study on parent-child estrangement. Individuals who were interested were directed to an end-to-end-encrypted online survey software (Jotform), where they could read information about the study, sign a consent form, and begin completing several questionnaires related to parent-child estrangement. The survey was available to complete for 6 months. These procedures resulted in 582 members of the online estrangement group completing the survey (response rate = ~30%).

To further understand and analyze the clinical findings, approval for secondary data analysis was received from the Human Research Ethics Board at the University of the Fraser Valley (101643), and the anonymized dataset was provided to the first and third authors for analysis.

### 2.2 Measures

Participants completed questionnaires in the Fall of 2024 using an online survey platform (JotForm) that is end-to-end encrypted. The original survey included five primary sections: (1) Demographics; (2) Estrangement Indicator; (3) The Big Five Aspects Scale (BFAS, DeYoung et al., 2007); (4) The Self-Compassion Scale (SCS, Neff, 2003); and (5) The Interpersonal Reactivity Index (IRI; Davis, 1983). Demographic characteristics included age, gender, sexual orientation, country of residence, marital status, education level and race/ethnicity. All questions in this survey were optional; therefore, not all respondents answered every question. To address the research question, the present study focused on demographic, estrangement, and personality data for this analysis.

#### 2.2.1 Estrangement indicator

Inspired by Reczek et al.'s (2022); Gilligan et al.'s (2015), and Becker and Hank (2022) approaches to construct a quantitative measure of parent-child estrangement, we constructed our binary outcome variable (0 = not estranged; 1 = estranged) using two variables: (1) how often the participant sees their parent or child (e.g., not at all, about once a year or less, several times a year, about once a month, two or three times a month, once a week, several times a week, or every day), and (2) how often the participant talks to their parent or child, via telephone, e-mail, text message, etc., (e.g., not at all, about once a year or less, several times a year, about once a month, two or three times a month, once a week, several times a week, or every day). Participants were categorized as being estranged from their parent or child if they reported: (1) seeing their family member “not at all” or “about once a year or less” and talking to them “not at all” or “about once a year or less.” Participants were categorized as not estranged (i.e., in a strained parent-child relationship) if they reported any other configuration of seeing and/or talking to their family member. Additionally, parents and adult children were asked if they willingly initiated the estrangement, using a dichotomous variable (0 = yes; 1 = no).

#### 2.2.2 Big five aspects scale (BFAS)

Participant personality was assessed using the Big Five Aspects Scale (BFAS; DeYoung et al., 2007), an empirically derived instrument for measuring the Big Five dimensions of personality, including their lower-level aspects: Extraversion (enthusiasm and assertiveness), Conscientiousness (industriousness and orderliness), Agreeableness (politeness and compassion), Neuroticism (withdrawal and volatility), and Openness (openness and intellect). The BFAS consists of 100 descriptions (e.g., “See myself as a good leader”; “Like to do things for others”) with which participants rate their agreement with how well each statement describes them using a five-point Likert-scale ranging from *strongly disagree* to *strongly agree*. The BFAS demonstrates strong internal reliability (mean α = 0.83) and test-retest reliability (mean *r* = 0.81) (DeYoung et al., 2007). In the present study, the BFAS demonstrated adequate internal reliability across subscales (mean α = 0.83). The BFAS has been validated against the Big Five Inventory (BFI) and the Revised NEO Personality Inventory (NEO PI-R), two of the most widely used instruments that measure personality. The BFAS also demonstrates adequate discriminant validity between the aspects within each domain, such that they do not lead to collinearity when used as simultaneous predictors in a logistic regression.

#### 2.2.3 Covariates

Demographic and social life information about parents and adult children was collected to analyze differences between estranged and non-estranged parents and adult children, and to adjust for characteristics that may confound the relationship between personality traits and estrangement (Reczek et al., 2022). In our models estimating estrangement in parents, we adjusted for parental gender (0 = “male”; 1 = “female”) to account for not only the differences between maternal and paternal estrangement (Becker and Hank, 2022; Kim, 2006; Pillemer, 2020; Reczek et al., 2022), but also gender differences in personality traits (Weisberg et al., 2011). We also adjusted for ethnicity (0 = “White”; 1 = “Hispanic”; 2 = “Asian”; 3 = “Multiracial”; 4 = “Other”) because of the association between ethnicity and the likelihood of estrangement (Reczek et al., 2022). From these five categories, we constructed a dichotomous ethnicity variable categorizing parents as either “White” or “Other” (comprising of those who identified as Asian, Multiracial, and/or Other) which was done to ensure a sufficient sample size of this category. Further, due to the relationship between educational attainment and estrangement (Reczek et al., 2022), along with education and certain personality traits (Poropat, 2009), we adjusted for parental educational attainment (0 = “less than high school”; 1 = “high school education”; 2 = “some college/diploma”; 3 = “bachelor's degree or equivalent”; and 4 = “Master's or PhD”). Parental marital status (0 = “single”; 1 = “married”; 2 = “divorced”) was also adjusted for, given the link between parental divorce and subsequent parent-child estrangement, perhaps through increased familial conflict and decreased contact between parents and their adult children (Becker and Hank, 2022). We also adjusted for parental age (in years), given the reduction in estrangement associated with older parental age (Schoppe-Sullivan et al., 2023), as well as the changes associated with personality and aging (Weisberg et al., 2011).

Models estimating estrangement in adult children were generally comparable because they shared the same basic associations across variables. We adjusted for adult-children's gender (0 = “male”; 1 = “female”) to account for gender differences in estrangement and personality traits. Due to differing cultural norms and family patterns associated with estrangement (Goldman and Cornwell, 2018; Reczek et al., 2022), we adjusted for adult child's ethnicity (0 = “White”; 1 = “Hispanic”; 2 = “Asian”; 3 = “Multiracial”; 4 = “Other”). From these five categories, we constructed a dichotomous ethnicity variable categorizing adult children as either “White” or “Other” (comprising of those who identified as Asian, Multiracial, and/or Other), which was done to ensure a sufficient sample size for this category. Adult children's sexual identity (0 = “straight”; 1 = “lesbian or gay”; 2 = “bisexual”; 3 = “other”) was also included due to the increased risk for lesbian, gay, and bisexual (LGB) adult children experiencing estrangement from parents compared to heterosexual children (Reczek et al., 2022; Stacey, 2021). Since the majority of our participants rated themselves as “straight,” we constructed a dichotomous sexuality variable categorizing adult children as either “straight” or “other” (comprising of participants who identified as lesbian or gay, bisexual, and/or other), to ensure a sufficient sample size for this category. As stated above, we also adjusted for adult-children's educational attainment (0 = “less than high school”; 1 = “high school education”; 2 = “some college/diploma”; 3 = “bachelor's degree or equivalent”; and 4 = “Master's or PhD”) due to its relationship with certain personality traits (Poropat, 2009). We also adjusted for adult children's marital status (0 = “single”; 1 = “married”; 2 = “divorced”) due to the association between life transitions such as marriage or divorce and subsequent estrangement from parents (Birditt et al., 2010; Reczek et al., 2022). Furthermore, given the relationship between an adult child's age and their relationship quality with their parents (Scheinfeld and Worley, 2018), we adjusted for the age of the adult child (in years).

### 2.3 Data analysis

All statistical analyses were computed utilizing SPSS-29.0 software (SPSS Inc., Chicago, IL, USA), and significance was set at *p* < 0.05. Descriptive statistics were analyzed separately for parents and adult children. Demographic variables were assessed with *t*-tests or Chi-squared tests to assess group differences between estranged and non-estranged parents and adult children. For our main analysis, we estimated a series of logistic regression models to predict estrangement as a function of Big Five personality traits. Subsequent logistic regression models were used to predict estrangement as a function of the 10 aspects of the Big Five traits, providing a more granular understanding of how personality may differ between estranged and non-estranged parents and adult children. All logistic regression models included the covariates listed above to test for confounding effects.

#### 2.3.1 Missing data

Listwise deletion was used to handle missing data. Of the 62 omitted cases, two participants had missing values for a variable indicating whether they were a parent or a child. Of the remaining omitted cases, 46 parents were omitted from analyses due to missing values in one of the estrangement indicator variables or the age variable. Of the adult children, 14 were omitted due to missing values in one of the estrangement indicator variables or the age variable. Based on these participant omissions, our new total sample included *n* = 520 (parents *n* = 368, adult children *n* = 152).

## 3 Results

### 3.1 Descriptive statistics

All participants in this study resided in the U.S. Using the operational definition of estrangement described above, of the 368 parents in this study, 48% (*N* = 191; 20 male, 171 female) were classified as estranged from at least one child, all of whom did not willingly initiate the estrangement, and 52% (*N* = 177 parents; 5 male, 172 female) were classified as non-estranged, currently engaging in strained dynamics with at least one child. All parents ranged in age from 39 to 80 (M = 59.81, SD = 7.63). The majority of parents were classified as White (95%), Married (57%), and Straight (96%). Of the 152 adult children in this study, 35% (*N* = 53; 9 male, 44 female) were classified as estranged from at least one parent, all of whom willingly initiated the estrangement, and 65% (*N* = 99; 11 male, 88 female) were classified as non-estranged, currently engaging in strained dynamics with at least one parent. All adult children ranged in age from 20 to 65 (M = 38.66, SD = 9.57). The majority of adult children were classified as White (82%) and Straight (81%).

Table 1 presents descriptive statistics for gender, race/ethnicity, educational attainment, marital status, sexuality, and age for estranged and non-estranged parents and adult children, analyzed separately. No statistical differences were found between estranged and non-estranged parents on the variables of ethnicity, educational attainment, marital status, sexuality, or age. A statistically significant difference was, however, found for gender, where estranged parents were disproportionately women, whereas men were more commonly represented in the non-estranged group, *X*^2^ (1, *N* = 368) = 8.48, *p* = 0.004. For estranged and non-estranged adult children, there were no statistical differences on the variables of gender, ethnicity, marital status, sexuality, or age. However, there was a statistically significant difference between estranged and non-estranged adult children on educational attainment, *X*^2^ (3, *N* = 152) = 7.99, *p* = 0.046. As shown in Table 1, estranged adult children were more likely to report a high-school level education and less likely to report postgraduate education compared to non-estranged adult children. In contrast, non-estranged adult children were more highly represented among those with master's or doctoral degrees.

**Table 1:** Demographic characteristics.

Characteristics	Parent *(n* = 368)		Adult child (*n* = 152)	
	Not estranged (*n =* 191) *N* (%)	Estranged (*n =* 177) *N* (%)	Not estranged (*n =* 99) *N* (%)	Estranged (*n =* 53) *N* (%)
Gender^*^				
Man	20 (10.5)	5 (2.8)	11 (11.1)	9 (17.0)
Woman	171 (89.5)	172 (97.2)	88 (88.9)	44 (83.0)
Race/ethnicity				
White	177 (92.7)	171 (96.6)	81 (81.8)	44 (83.0)
Other	14 (7.3)	6 (3.4)	18 (18.2)	9 (17.0)
Education^*^				
Less than high school	3 (1.6)	3 (1.7)	0 (0)	0 (0)
High school	28 (14.7)	29 (16.4)	14 (14.1)	14 (26.4)
Some college/diploma	23 (12.0)	22 (12.4)	21 (21.2)	8 (15.1)
Bachelors or equivalent	92 (48.2)	67 (37.9)	33 (33.3)	23 (43.4)
Masters or PhD	45 (23.6)	56 (31.6)	31 (31.3)	8 (15.1)
Marital status				
Single	19 (9.9)	27 (15.3)	40 (40.4)	18 (34.0)
Married	115 (60.2)	96 (54.2)	46 (46.5)	26 (49.1)
Divorced	57 (29.8)	54 (30.5)	13 (13.1)	9 (17.0)
Sexuality				
Straight	183 (95.8)	171 (96.6)	79 (79.8)	44 (83.0)
Other	8 (4.2%)	6 (3.4)	20 (20.2)	9 (17.0)
**Age**	59.37 (8.32 SD)	60.29 (6.80 SD)	40.02 (10.16 SD)	38.98 (8.41 SD)

* *p* < 0.05. Group differences were assessed using chi-square tests of independence for categorical variables and independent-samples *t*-tests for age. Among parents, gender was significantly associated with estrangement status, χ^2^(1, *N* = 368) = 8.48, *p* = 0.004. Among adult children, educational attainment was significantly associated with estrangement status, χ^2^(3, *N* = 152) = 7.99, *p* = 0.046. No other demographic variables were significantly associated with estrangement status in either group.

### 3.2 Main analyses

A series of binary logistic regression analyses were conducted separately for parents and adult children to examine whether Big Five personality traits and their 10 aspects were associated with estrangement status while controlling for relevant demographic covariates. Across all models, gender, race/ethnicity, educational attainment, marital status, sexuality (adult children only), and age were included as covariates.

Results from the logistic regression models examining the Big Five traits are presented in Table 2. Among parents, extraversion was a significant predictor of estrangement, with higher levels associated with a 79% increase in the odds of estrangement (*B* = 0.58, *SE* = 0.27, OR = 1.79, *p* = 0.031). None of the other Big Five traits, namely agreeableness, conscientiousness, neuroticism, or openness, were significantly associated with estrangement among parents. With respect to demographic covariates, women were significantly more likely than men to report estrangement (*B* = 1.20, SE = 0.55, OR = 3.34, *p* = 0.027), indicating that women had more than three times the odds of estrangement compared to men, after controlling for all other variables.

**Table 2:** Logistic regression results predicting estrangement among parents and adult children from Big Five personality traits.

Variable	Parent			Adult child		
	B	SE B	OR	B	SE B	OR
*Covariates* gender (man: reference)						
Woman	1.20	0.55	3.34^*^	−0.95	0.60	0.39
Race/ethnicity (white: reference)						
Other	−0.98	0.53	0.38	−0.17	0.54	0.85
Education (less than high school: reference)						
High school	0.22	0.90	1.24	–	–	–
Some college/diploma	0.10	0.90	1.10	−0.90	0.64	0.41
Bachelors or equivalent	−0.13	0.87	0.88	−0.23	0.53	0.80
Masters or PhD	0.34	0.88	1.41	−1.29	0.60	0.28^*^
Marital status (single: reference)						
Married	−0.61	0.36	0.54	0.53	0.47	1.69
Divorced	−0.44	0.38	0.65	1.19	0.68	3.27
Sexuality (straight: reference)						
Other	–	–	–	−0.20	0.52	0.82
**Age**	0.22	0.02	1.02	−0.04	0.03	0.97
Personality						
Agreeableness	0.12	0.30	1.13	0.79	0.51	2.19
Conscientiousness	−0.06	0.25	0.94	−0.07	0.44	0.93
Extraversion	0.58	0.27	1.79^*^	0.12	0.41	1.12
Neuroticism	0.05	0.19	1.05	−0.22	0.32	0.80
Openness	−0.04	0.27	0.96	−1.53	0.52	0.22^**^

* *p* < 0.05;

** *p* < 0.01. The reference group for the education variable among adult children was high school, since no participant had less than a high school education.

Among adult children, openness was the only Big Five trait that significantly predicted estrangement. Higher openness was associated with a 78% reduction in the odds of estrangement (*B* = −1.53, *SE* = 0.52, OR = 0.22, *p* = 0.003). No other Big Five traits significantly predicted estrangement in the adult-child model. In terms of demographic covariates, adult children with a master's or doctoral degree had a 72% reduction in the odds of estrangement compared to those whose highest level of education was high school (*B* = −1.23, SE = 0.60, OR = 0.28, *p* = 0.03). Gender, race/ethnicity, marital status, sexuality, and age were not significant predictors in the adult-child model.

Results from the aspect-level logistic regression analyses are presented in Table 3. Among parents, higher enthusiasm, which is a facet of extraversion, was associated with a 57% increase in the odds of estrangement (*B* = 0.45, *SE* = 0.22, OR = 1.57, *p* = 0.039). No other personality aspects significantly predicted estrangement among parents.

**Table 3:** Logistic regression results predicting estrangement among parents and adult children from the ten aspects of the Big Five personality traits.

Variable	Parent			Adult child		
	B	SE B	OR	B	SE B	OR
Covariates Gender (man: reference)						
Woman	1.17	0.55	3.22^*^	−1.14	0.62	0.32
Race/ethnicity (white: reference)						
Other	−0.97	0.54	0.38	−0.24	0.54	0.79
Education (less than high school: reference)						
High school	0.20	0.90	1.23	–	–	–
Some college/diploma	0.04	0.91	1.04	−0.95	0.66	0.39
Bachelors or equivalent	−0.18	0.88	0.83	−0.12	0.55	0.89
Masters or PhD	0.25	0.89	1.28	−1.17	0.62	0.31
Marital status (Single: reference)						
Married	−0.60	0.36	0.55	0.66	0.49	1.93
Divorced	−0.43	0.38	0.65	1.07	0.72	2.91
Sexuality (straight: reference)						
Other	–	–	–	−0.16	0.54	0.85
**Age**	0.02	0.02	1.02	−0.04	0.03	0.96
Personality						
**Agreeableness**						
Compassion	0.09	0.32	1.09	0.20	0.64	1.22
Politeness	−0.09	0.32	0.91	0.38	0.46	1.46
Conscientiousness						
Industriousness	0.24	0.30	1.27	−0.28	0.49	0.76
Orderliness	−0.19	0.24	0.83	0.11	0.38	1.12
Extraversion						
Enthusiasm	0.45	0.22	1.57^*^	0.02	0.39	1.02
Assertiveness	0.02	0.29	1.02	0.58	0.53	1.79
Neuroticism						
Withdrawal	0.16	0.24	1.18	0.62	0.49	1.87
Volatility	−0.01	0.22	0.99	−0.77	0.40	0.46
Openness						
Intellect	0.07	0.25	1.07	−0.93	0.40	0.40^*^
Openness	−0.09	0.25	0.91	−0.85	0.39	0.43^*^

* *p* < 0.05. The reference group for the education variable among adult children was high school, since no participant had less than a high school education.

Among adult children, two openness-related aspects were significant predictors of estrangement. Higher levels of intellect were associated with a 60% reduction in the odds of estrangement (*B* = −0.93, *SE* = 0.40, OR = 0.40, *p* = 0.020). Similarly, higher openness was associated with a 57% reduction in the odds of estrangement (*B* = −0.85, *SE* = 0.39, OR = 0.43, *p* = 0.027). No aspects of agreeableness, conscientiousness, extraversion, or neuroticism were significantly related to estrangement among adult children, and none of the demographic covariates reached statistical significance in this model.

## 4 Discussion

In the present study, we aimed to determine whether trait personality predicted estrangement outcomes among non-matched parents and adult children experiencing strained parent-child dynamics. Broadly, results indicate that personality traits significantly predict estrangement outcomes, though the nature of that role differs for parents and adult children. The major findings include: (1) Higher levels of trait extraversion, and its facet, enthusiasm, significantly predicted increased odds of estrangement among parents; (2) Higher trait openness, and both its facets, intellect and openness, significantly predicted decreased odds of estrangement among adult children. With respect to demographic covariates, among parents, mothers were significantly more likely to be estranged than fathers. Additionally, among adult children, those whose highest level of education was a master's or doctoral degree were significantly less likely to be estranged compared to those who had only completed a high school education.

The finding that higher parental extraversion was associated with increased odds of being estranged by one's adult child can be understood within broader trait research on interpersonal functioning. Although trait extraversion is often associated with social engagement, positive affect, and interpersonal warmth (Depue and Collins, 1999), it is also linked to interpersonal dominance, assertiveness, and impulsivity (Deng et al., 2021; Gómez-Bujedo et al., 2020; Roberts et al., 2006). Prior research further indicates that extraversion is associated with relationship conflict (Bono et al., 2002), antagonistic interaction patterns (Volk et al., 2021), and forceful conflict strategies that emphasize control over mutual accommodation (Grant et al., 2011; Park and Antonioni, 2007), with a recent meta-analysis (Tehrani and Yamini, 2020) suggesting a tendency toward more forceful expression and win-lose orientation in conflict. However, given that the facet of enthusiasm, rather than assertiveness, was the significant predictor at the aspect level, these findings suggest that estrangement risk associated with parental extraversion may not be limited to dominance and control. The aspect of enthusiasm is characterized by sociability, positive emotionality, and a strong orientation toward social engagement and reward, with individuals high on this facet seeking frequent interactions and expressing affect in an energetic, outwardly engaged manner (Ashton et al., 2002; DeYoung et al., 2007; Smillie, 2013). Consistent with research on interpersonal fit, discrepancies between individuals' preferred and experienced levels of closeness are associated with poorer relationship quality, even when overall levels of closeness are high (Frost and LeBlanc, 2022). Therefore, even well-intentioned expressions of warmth or connection may be experienced as misattuned when they fail to align with the adult child's needs, which may potentially contribute to relational strain (Rice et al., 2020).

In the context of estrangement research, these interpersonal tendencies may begin to clarify why higher parental extraversion is associated with an increased risk of estrangement. Reczek (2025) emphasized that a central motivation for estrangement among adult children is a perceived lack of mutual respect from their parents. Interaction patterns associated with assertiveness, such as hierarchical authority, forceful expression of opinions, and reduced accommodation, closely map onto these concerns and may thereby undermine an adult child's expectations of mutual respect. At the same time, enthusiasm-related tendencies may represent a distinct pathway to similar outcomes. For instance, parents high in enthusiasm may express warmth through frequent and emotionally expressive contact, yet may struggle to calibrate that expressiveness in response to the adult child's need for space or emotional attunement. In relationships already characterized by tension, frequent and emotionally expressive bids for connection may be experienced not as care but as relational pressure, particularly when the adult child prioritizes autonomy, accountability, or emotional safety over increased contact. In this way, enthusiasm-related tendencies may inadvertently undermine the conditions that adult children associate with mutual respect and emotional safety. Consistent with this interpretation, qualitative work indicates that adult children often attribute estrangement to parents perceived as attention-seeking, demanding, or overly focused on their own relational needs (Agllias, 2016). Taken together, these findings suggest that higher parental extraversion may increase estrangement risk through interpersonal styles characterized by dominance and connection-oriented pathways-that, in different ways, may conflict with adult children's expectations for mutual respect, autonomy and emotional safety.

The finding that higher trait openness among adult children was associated with decreased odds of initiating estrangement can likewise be understood within the broader personality literature. Trait openness is associated with intellectual curiosity, cognitive flexibility, tolerance for ambiguity, acceptance of others, and receptivity to novel perspectives (Abu Raya et al., 2023; DeYoung et al., 2010; McCrae and Costa, 1983). Individuals high in openness tend to exhibit greater mental flexibility by adapting their ways of thinking and behaving to changing contexts, allowing them to be more adaptable in uncertain and complex situations (Abu Raya et al., 2023). Moreover, trait openness is associated with reduced prejudice and greater ideological tolerance (Ng et al., 2021), as well as an increased ability to manage and adapt to conflicting cultural contexts and values (Abu Raya et al., 2023; Schwaba et al., 2018). Research on conflict management indicates that trait openness is associated with compromise and integrative conflict styles (Tehrani and Yamini, 2020), creative problem solving, exploration of alternative interpretations, and reduced reliance on rigid adherence to one's beliefs (Abu Raya et al., 2023). In fact, Park and Antonioni (2007), in their exploration of Big Five personality traits and conflict resolution, state that trait openness is particularly critical for constructive conflict resolution, and that people high in trait openness are more receptive to others' perspectives, more accommodating, less competing, and more likely to see opportunities for collaboration rather than win-lose outcomes.

When examined in light of estrangement research, these dispositional tendencies help clarify why higher trait openness may be associated with reduced odds of initiating estrangement among adult children. For instance, a study by Agllias (2015) exploring the experiences of estranged parents found that many participants reported that their adult child's inability to incorporate, integrate, and manage the tension between differing perspectives was a key factor they named in explaining their child's decision to initiate estrangement. This account aligns with Reczek (2025) analysis of adult children, which emphasizes that estrangement is often motivated by perceived violations of accountability, mutual respect, intentional time, and emotional and physical safety. From this perspective, adult children high in trait openness may be less inclined to interpret such violations as warranting relational rupture, in large part because they are more able to exhibit greater cognitive flexibility and tolerance for value differences. Trait openness may therefore help contextualize previous research suggesting that some adult children respond to value differences by initiating estrangement, while others, who experience similar family differences, respond with continued engagement (Gilligan et al., 2015). In this way, trait openness may help explain why some adult children maintain relationships under conditions that may lead others to estrangement.

With respect to demographic variables, several findings warrant consideration. Contrary to much of the existing literature, parental divorce did not significantly predict estrangement. Prior research has frequently identified divorce as a risk factor for parent-child estrangement, often through mechanisms such as reduced contact, interparental conflict, and shifting family alliances (Becker and Hank, 2022; Pillemer, 2020; Reczek et al., 2022). However, the absence of this effect in the present study is likely attributable to the characteristics of our sample; all participants were drawn from strained or estranged family contexts, thereby restricting variability in relational functioning and reducing contrast with intact, low-conflict families. In such a sample, circumstantial stressors like divorce may be relatively common across both estranged and non-estranged groups, diminishing their predictive utility.

Educational attainment emerged as a significant predictor of estrangement among adult children, with those holding master's or doctoral degrees demonstrating reduced odds of estrangement compared to those with only a high school education. This finding is consistent with research linking higher education to greater cognitive complexity, problem-solving, and critical thinking skills (Jaramillo Gómez et al., 2025; Poropat, 2009), which may facilitate sustained engagement despite relational strain. Alternatively, higher education has been consistently linked to greater financial security, occupational stability, and access to social resources (Hout, 2012), which are factors that prior work has identified as life course influences on the likelihood and timing of estrangement decisions (Reczek et al., 2022; Stee, 2022). This association is consistent with family stress theory, which holds that stressors within the family system are often buffered by family resources, such as finances. Chronic financial stress is a well-documented source of interpersonal conflict and family strain (Neppl et al., 2016; Stee, 2022), and individuals with fewer economic resources may be more vulnerable to the accumulating relational tensions that increase the risk of estrangement. When such stressors compound and resources are insufficient to absorb them, the family system can become destabilized, potentially culminating in significant conflict or estrangement. By contrast, the material and psychological buffers associated with higher socioeconomic status may reduce overall family conflict and provide individuals with the stability needed to invest in repairing and maintaining strained relationships rather than severing them. As such, to the extent that educational attainment serves as a proxy for socioeconomic status, it may buffer the compounding stressors that precipitate relational rupture through its material and psychological resources. Importantly, these explanations are not mutually exclusive, and future research would benefit from directly examining whether cognitive complexity or socioeconomic resources mediate the association between educational attainment and estrangement.

Finally, mothers were significantly more likely than fathers to be represented in the estranged parent group; however, this finding should be interpreted with caution, given the sample's composition. Approximately 93% of the parent participants identified as women, resulting in limited representation of fathers to make meaningful gender comparisons. As such, the observed gender difference is likely an artifact of recruitment rather than evidence that mothers are inherently more likely to experience estrangement. The overrepresentation of mothers may also reflect broader patterns observed in estrangement support communities, as seen in a study by Blake et al. (2022), which reflected a similar pattern of a disproportionate number of mothers compared to fathers in an online estrangement support group.

### 4.1 Limitations and future directions

Several limitations of the present study warrant consideration. First, reliance on self-report measures may reflect how parents and adult children perceive and report on themselves rather than how they are perceived by their respective family members or by their behavioral tendencies. Moreover, parents and adult children in this study were not matched dyadically, precluding examination of interactive personality effects within actual family units. Because estrangement unfolds within a relational system, future research using matched parent-child pairs would allow for investigation of personality configurations and reciprocal dynamics. Such designs could also incorporate informant or peer-reports to reduce shared method bias. However, such a study may be particularly difficult to accomplish due to the nature of estrangement and the limited contact between the parties.

Second, the cross-sectional design precludes causal inference. Although FFT conceptualizes personality traits as relatively stable basic tendencies that shape downstream relational outcomes, the present data cannot establish temporal direction. It remains possible that the experience of estrangement influences how individuals report their personality, particularly on self-report measures. Longitudinal research assessing personality prior to estrangement onset would provide stronger tests of the directional pathways implied by FFT.

Third, the sample was non-representative in several respects. Participants were recruited from a single online estrangement support community, introducing potential self-selection bias and limiting generalizability. The sample was predominantly White, female, straight, and located in the United States. These characteristics constrain the extent to which findings can be generalized to fathers, racially and ethnically diverse families, LGBTQ+ family members, or individuals embedded in non-Western cultural contexts where norms surrounding family obligation may differ substantially from the democratized kinship repertoire described by Reczek (2025).

Finally, although McCrae and Sutin (2018) argue that examining trait-level predictors of behavioral outcomes without directly measuring mediating characteristic adaptations is a theoretically justified and practical starting point, the present study does not permit precise identification of the mechanisms linking personality traits to estrangement. FFT maintains that traits influence behavior through characteristic adaptations, such as relational expectations, coping strategies, conflict styles, and interpretive frameworks (McCrae and Sutin, 2018). Establishing that traits predict estrangement therefore provides an initial empirical foundation, but future research must move toward specifying mediation pathways that allow for more precise mapping of how basic tendencies translate into estrangement decisions. For example, testing whether endorsement of democratized kinship attitudes mediates the relationship between openness and estrangement initiation would meaningfully extend the present findings.

### 4.2 Conclusion

Taken together, the present findings underscore the need to consider not only external influences but also personality traits in understanding parent-child estrangement. Specifically, the present findings are consistent with the propositions within FFT. That is, higher parental trait extraversion may be associated with characteristic adaptations related to one's communication style, conflict tendencies, and needs for recognition, which may be experienced by adult children as controlling or misaligned with expectations for autonomy and mutual respect. In contrast, higher trait openness among adult children may be associated with characteristic adaptations characterized by cognitive flexibility, tolerance of difference, and integrative conflict strategies, potentially reducing the likelihood that relational tensions culminate in estrangement. From an FFT perspective, estrangement may therefore be understood not simply as a reaction to family conflict or cultural change, but as the outcome of long-term interactions among basic tendencies, culturally shaped characteristic adaptations, and situational triggers. In this way, the findings extend circumstantial and cultural accounts of estrangement by identifying personality as a theoretically grounded and previously unexamined component. Therefore, the present study lays the groundwork for future research examining the specific processes through which traits are associated with estrangement. More broadly, this work contributes to a more integrative understanding of parent-child estrangement that bridges relational experiences, cultural frameworks, and enduring personality structure.

## Data Availability

The raw data supporting the conclusions of this article will be made available by the authors, without undue reservation.
